# Sinus Venosus Atrial Septal Defect as an Overlooked Source of Shortness of Breath Among Patients With Pulmonary Arterial Hypertension

**DOI:** 10.7759/cureus.62935

**Published:** 2024-06-22

**Authors:** Sonia Vicenty-Rivera, Victor H Molina-Lopez, Porfirio E Diaz-Rodriguez, Luis A Molinary-Jimenez

**Affiliations:** 1 Cardiology, Bruce W. Carter Department of Veterans Affairs Medical Center, Miami, USA; 2 Cardiology, Veterans Affairs Caribbean Healthcare System, San Juan, PRI

**Keywords:** intracardiac shunt, pulmonary hypertension, partial anomalous pulmonary venous connection, partial anomalous pulmonary venous return, sinus venosus atrial septal defect

## Abstract

Sinus venosus atrial septal defects (SVASD) associated with partial anomalous pulmonary venous return (PAPVR) can be overlooked as a source of dyspnea in adult patients with pulmonary hypertension. We present the case of a 61-year-old male with exertional dyspnea initially attributed to pulmonary hypertension, who was subsequently diagnosed with SVASD and right superior PAPVR. This case underscores the critical importance of maintaining high clinical awareness and utilizing multimodal imaging techniques in cardiology to accurately diagnose and manage pulmonary hypertension secondary to congenital heart disease. Timely surgical correction can significantly improve morbidity and mortality outcomes.

## Introduction

Congenital heart disease (CHD) is the most common congenital condition in the United States [1]. Among adults, atrial septal defect (ASD) is one of the most common CHDs, with an incidence of 0.13% in the USA [2]. It is among the most frequently diagnosed causes of non-cyanotic CHD in adults [3-4]. There are four types of ASDs, with the ostium secundum defect being the most common. Sinus venosus ASD (SVASD) constitutes 5-6% of ASDs, with most cases associated with partial anomalous pulmonary venous return (PAPVR). The most common types of PAPVR are usually the right upper and right middle pulmonary veins connecting to the junction of the superior vena cava (SVC) and right atrium (RA) [5].

SVASD with PAPVR is an often-overlooked diagnosis in patients presenting with pulmonary arterial hypertension. Common conditions can easily mask these congenital defects, leading to delayed or missed diagnoses. This case report underscores the critical importance of comprehensive multimodality imaging and heightened clinical awareness in identifying these subtle yet significant anomalies. Timely and accurate diagnosis, followed by appropriate surgical intervention, can significantly improve patient outcomes, reducing symptoms and decreasing mortality risk due to right heart failure and Eisenmenger physiology [1-5]. This case emphasizes the importance of routine screening for CHD, including SVASD, in patients with unexplained dyspnea and pulmonary arterial hypertension, ensuring optimal management and care.

## Case presentation

A 61-year-old male patient was evaluated for progressively worsening dyspnea on exertion. His clinical history included obstructive sleep apnea, stage IIIA chronic kidney disease, bronchial asthma, and idiopathic pulmonary hypertension previously treated at another institution. Despite pulmonary vasodilator therapy (Abrisentan 10 mg daily), his shortness of breath had worsened over the past six months. Symptoms included dyspnea with mild to moderate exertion and when bending forward. A six-minute walk test (6MWT) showed a maximal distance of 453 meters. The pulmonary function test was normal. Previous evaluations for pulmonary hypertension included right heart catheterization, which indicated pre-capillary pulmonary hypertension with mean pulmonary artery pressure (mPAP) of 28 mmHg, a pulmonary capillary wedge pressure (PCWP) of 17 mmHg, diastolic pressure gradient (DPG) of 3 mmHg, and central venous pressure (CVP) of 15 mmHg.

**Figure 1:**
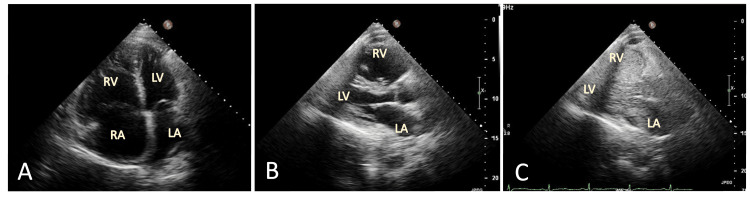
Initial TTE evaluation (A) Apical 4-chamber view. (B) Parasternal long axis view. (C) Demonstrating markedly enlarged right heart structures with marked right-to-left shunting of agitated saline. TTE: transthoracic echocardiogram; RV: right ventricle; RA: right atrium; LV: left ventricle; LA: left atrium.

The patient’s chest radiography revealed prominence and engorgement of the perihilar vascular structures and interlobar pulmonary arteries. A twelve-lead electrocardiogram showed right ventricular hypertrophy. A transthoracic echocardiogram (TTE) demonstrated systolic and diastolic septal flattening consistent with right ventricular (RV) volume and pressure overload, right atrial and ventricular enlargement, right ventricular hypertrophy, and elevated noninvasive right heart pressures (Figure 1A, 1B). CVP was estimated at 15 mmHg, and right ventricular systolic pressure (RVSP) at 40 mmHg. No clear evidence of a patent foramen ovale (PFO) or communication between the right and left atria was found on color flow Doppler. However, an agitated saline injection revealed a large right-to-left shunt (Figure 1C).

**Figure 2:**
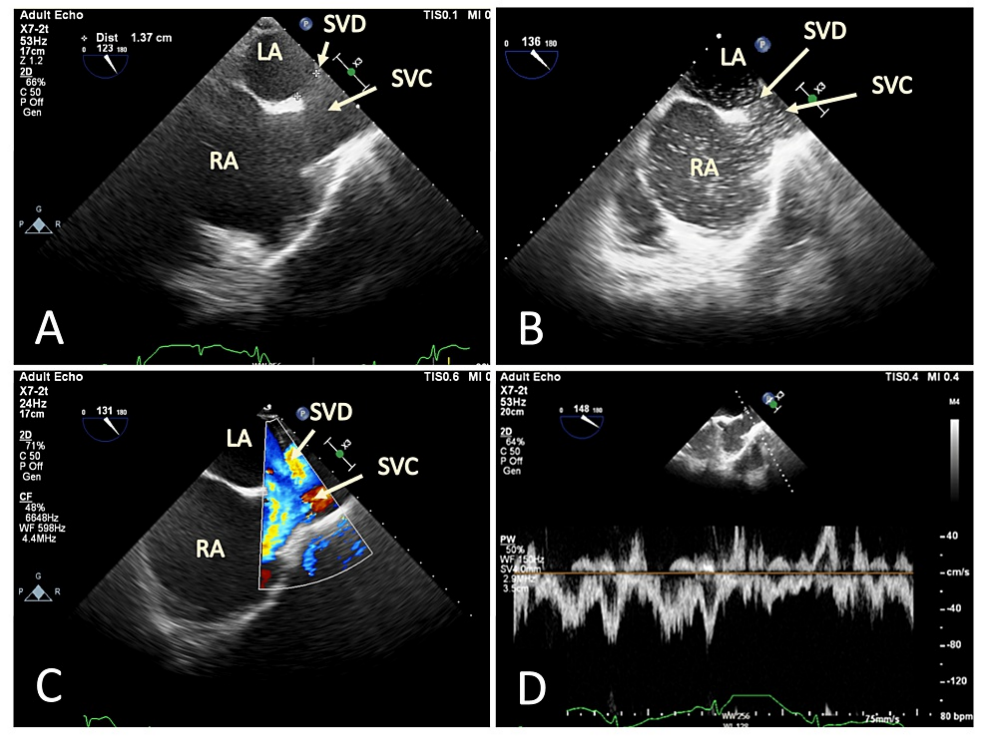
TEE evaluation of right-to-left shunt demonstrating the SVD with high flow concerning for an APVR (A) TEE of modified bicaval view demonstrating the large SVD. (B) With a marked amount of right-to-left shunt of agitated saline injected through an upper extremity peripheral vein. (C) Color flow doppler. (D) Pulse wave doppler (C and D demonstrate a bidirectional shunting and suggesting a predominant flow from the RUPV into the SVC). TEE: transesophageal echocardiogram; RV: right ventricle; RA: right atrium; LV: left ventricle; LA: left atrium; SVD: sinus venosus defect; SVC: superior vena cava; APVR: anomalous pulmonary venous return; RUPV: right upper pulmonary vein.

A transesophageal echocardiogram (TEE) was performed to delineate the cardiac defect better, confirming an atrial septal defect of the sinus venosus type (Figures 2A-2D).

**Figure 3:**
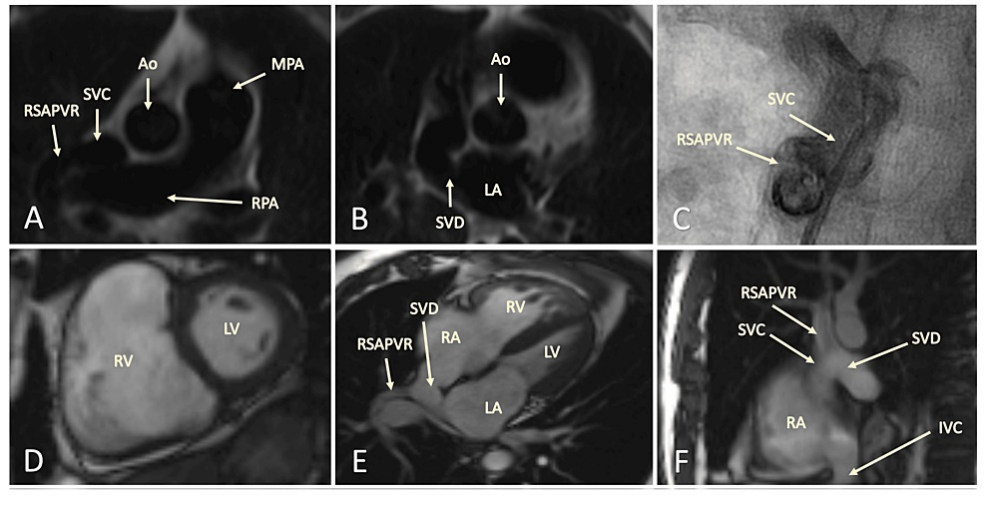
CMR images confirming diagnosis of SVD with RSAPVR (A-B) CMR T1 weighted images revealing the RSAPV joining the SVC and the SVASD. (C) Selective angiography of the RSAPV, SVC, and SVASD junction. (D) CMR T2 weighted images demonstrating severe dilation of the RV on diastole. (E-F) 4 chamber view depicting the junction of the RSAPVR, SVC, and SVD. CMR: cardiac magnetic resonance imaging; RV: right ventricle; RA: right atrium; LV: left ventricle; LA: left atrium; SVD: sinus venosus defect; SVC: superior vena cava; RSAPVR: right superior anomalous pulmonary venous return.

Given these findings, a cardiac MRI (CMR) was conducted, revealing a partial anomalous pulmonary venous return of the right upper pulmonary vein draining into the superior vena cava (SVC), with the anomalous connection located proximal to the SVC and right atrial junction, along with a sinus venosus ASD (Figure 3A-3F). 

Right heart catheterization showed a 17% step-up in blood oxygen saturation between the SVC and RA, with a pulmonary-to-systemic shunt ratio of 2.5 (QP:QS), and no indication of Eisenmenger's physiology (Table 1). Coronary angiography was remarkable for normal coronary arteries.

**Table 1:** Right heart pressures and hemodynamics

Pressure Values	
Right atrial mean pressure	15 mmHg
Right ventricular pressures	40/13 mmHg
Pulmonary artery pressures	35/17 mmHg, mean 25 mmHg
Pulmonary capillary wedge pressure	16 mmHg
Cardiac output	5.13 L/min
Pulmonary vascular resistance	1.8 Wood units
Systemic vascular resistance	16 Wood Units

**Figure 4:**
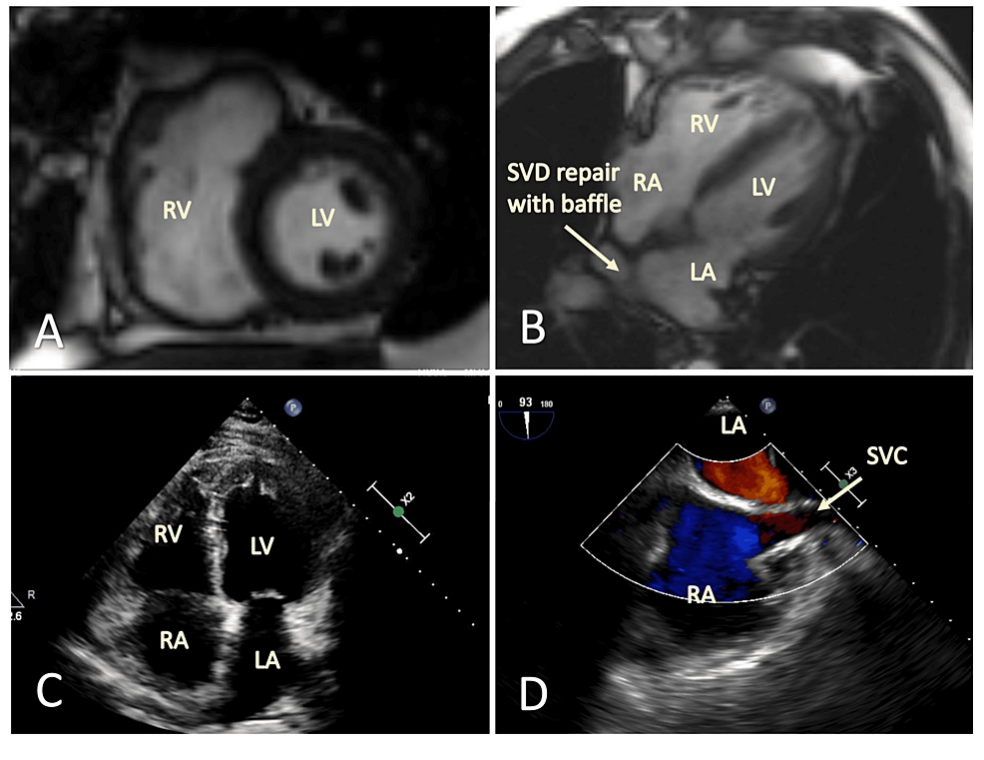
Follow-up images after surgical repair of the RSAPVR and SVD CMR images on follow-up; (A) Demonstrates an improvement in RV dilation. (B) The surgical correction of the SVD and RSAPVR repair with a baffle conduit can be appreciated, restoring the right superior pulmonary vein into the LA. (C) TTE images in the apical 4-chamber view demonstrate improved RV dilation. (D) TEE image of the bicaval mid-esophageal view demonstrates resolution of the inter-atrial shunt. CMR: cardiac magnetic resonance imaging; RV: right ventricle; RA: right atrium; LV: left ventricle; LA: left atrium; SVD: sinus venosus defect; SVC: superior vena cava; RSAPVR: right superior anomalous pulmonary venous return; TTE: transthoracic echocardiogram; TEE: transesophageal echocardiogram.

The patient underwent surgical correction with a baffle conduit and patch procedure and had an uneventful recovery, except for postoperative atrial fibrillation treated with Warfarin and Dofetilide. At a follow-up clinic evaluation one month post-surgery, he reported resolution of exertional dyspnea. Follow-up imaging studies with TTE and CMR showed a reduction in right heart chamber size, correction of the intracardiac shunt (Figure 4A-4D), and decreased right-sided pressures (RVSP 32 mmHg and CVP 3 mmHg). Additionally, the patient exhibited improved exercise capacity (6MWT increased from 453 meters to 510 meters) and better biomarker parameters (NT pro-BNP decreased from 407 to 37.64 pg/ml) at two months post-operatively.

## Discussion

CHD is the most common congenital condition, affecting approximately 1% or one in 120 live births in the USA [1]. Among adults, ASD is one of the most common CHDs, affecting 13 of every 10,000 individuals in the USA [2]. It is the most frequently diagnosed non-cyanotic CHD in adults, following bicuspid aortic disease and mitral valve prolapse, occurring in about 25-30% of adults with congenital heart failure [3-4]. Four types of ASDs are described in the literature, with the ostium secundum defect being the most common, accounting for 75% of cases. The sinus venosus type constitutes 5-6% of ASDs, and in 85% of these cases, it is associated with PAPVR [5]. In about 90% of cases, the right upper and middle pulmonary veins connect to the junction of the SVC and RA. Less commonly, inferior sinus venosus defects originate at the mouth of the IVC and extend into the LA, leaving no residual atrial septal tissue at the inferior margin.

The etiology of ASD development is unknown but is believed to involve an interplay of genetic predisposition, such as mutations in cardiac transcription factor genes NKX2-5, GATA4, and TBX5, and environmental risk factors, including maternal exposure to alcohol, cigarettes, antidepressants, and diabetes mellitus [6-10]. Surgical repair has historically been the primary management approach. SVASD was initially described by Peacock in 1858 and Waggstaffe in 1968 [11-12]. This type of defect encompasses approximately 5-10% of all ASDs. Unlike other ASDs, which have a 2:1 female-to-male ratio, SVASD has a 1:1 ratio [1-2]. During embryonic development, the sinus venosus incorporates into the right atrium wall, forming the sinus venarum [13]. SVASD occurs when tissue separating the right pulmonary veins is deficient, causing the pulmonary venous return to deviate to the right atrium.

SVASD can remain undiagnosed for years as symptoms may not present until adulthood, often manifesting as exercise intolerance and dyspnea. Other symptoms include arrhythmias, paradoxical emboli, and inadvertent passage of cardiovascular leads or catheters through the defect during interventions [14]. Patients can develop pulmonary hypertension due to left-to-right shunting, which is more pronounced in SVASD with PAPVR, potentially leading to an earlier onset of Eisenmenger physiology. TTE, while the gold standard for ASD diagnosis, has low sensitivity for SVASD due to the posterior location of the interatrial communication. SVASD with PAPVR is often misdiagnosed as primary pulmonary hypertension. Therefore, a high index of suspicion is necessary, especially in patients with unexplained right heart chamber dilation on TTE [15]. Accurate diagnosis typically requires a multi-imaging approach, including TEE, CMR, and cardiac CT [16-18].

Cardiac catheterization is valuable for assessing coronary artery disease in older patients and evaluating hemodynamic significance, though not directly for ASD diagnosis. An oxygen step-up of 10% in the right heart catheterization oximetry run indicates left-to-right shunting at the atrial level. Accepted indications for ASD closure include right-sided cardiac volume loading, symptomatic patients, those with exercise-related cyanosis, paradoxical thromboembolism, and prophylaxis for high-risk non-cardiac procedures. Surgical repair involves redirecting the anomalous pulmonary venous connection to the LA and is associated with low morbidity and mortality, even in older adults. However, postoperative atrial fibrillation is more common in older patients despite functional improvements [19]. Recently, transcatheter interventions using covered stents guided by multi-imaging have emerged as alternatives. A case series by Clément Batteux et al. demonstrated the feasibility and safety of this approach in selected patients, emphasizing the necessity of pre-procedural multimodality imaging with 3D modeling [20].

## Conclusions

SVASD with PAPVR can be an overlooked source of shortness of breath in patients diagnosed with pulmonary arterial hypertension. This case underscores the importance of maintaining a high index of suspicion for SVASD in patients presenting with unexplained dyspnea and right heart chamber dilation. Utilizing a multi-modality imaging approach, including TTE, TEE, and cardiac MRI, is essential for accurate diagnosis. Given the significant impact of timely and accurate diagnosis on patient outcomes, we recommend routine screening for SVASD in patients with newly diagnosed pulmonary arterial hypertension. Prompt identification and appropriate surgical intervention can lead to substantial improvements in symptoms and a decrease in mortality risk.
